# Population modifiable risk factors associated with under-5 acute respiratory tract infections and diarrhoea in 25 countries in sub-Saharan Africa (2014–2021): an analysis of data from demographic and health surveys

**DOI:** 10.1016/j.eclinm.2024.102444

**Published:** 2024-02-03

**Authors:** Kedir Y. Ahmed, Abel F. Dadi, Getiye Dejenu Kibret, Habtamu Mellie Bizuayehu, Tahir A. Hassen, Erkihun Amsalu, Daniel Bekele Ketema, Zemenu Yohannes Kassa, Meless G. Bore, Animut Alebel, Addisu Alehegn Alemu, Jemal E. Shifa, Cheru Tesema Leshargie, Subash Thapa, Syed Haris Omar, Allen G. Ross

**Affiliations:** a Rural Health Research Institute, Charles Sturt University, Orange, NSW 2800, Australia; b Menzies School of Health Research, Charles Darwin University, Darwin, Northern Territory, Australia; c Addis Continental Institute of Public Health, Addis Ababa, Ethiopia; d Faculty of Health, School of Public Health, University of Technology Sydney, Ultimo, NSW, Australia; e Centre for Health Systems and Safety Research, Australian Institute of Health Innovation, Faculty of Medicine, Health and Human Sciences, Macquarie University, Australia; f First Nations Cancer and Wellbeing (FNCW) Research Program, School of Public Health, The University of Queensland, Australia; g Center for Women’s Health Research, College of Health, Medicine and Wellbeing, The University of Newcastle, NSW, Australia; h Faculty of Medicine and Health, Sydney Medical School, University of Sydney, Australia; i St. Paul Hospital Millennium Medical College, Addis Ababa, Ethiopia; j The George Institute for Global Health, University of New South Wales (UNSW), Sydney, Australia; k School of Public Health, College of Medicine and Health Science, Debre Markos University, Ethiopia; l College of Medicine and Health Sciences, Hawassa University, Hawassa, Ethiopia; m School of Nursing and Midwifery, University of Technology Sydney, Sydney, Australia; n School of Nursing, College of Medicine and Health Science, Hawassa University, Hawassa, Ethiopia; o School of Women’s and Children’s Health, University of New South Wales Sydney, Kensington, Australia; p College of Medicine and Health Science, Debre Markos University, Ethiopia; q School of Dentistry and Medical Sciences, Charles Sturt University, Orange, NSW 2800, Australia

**Keywords:** Population attributable fractions, Acute respiratory tract infections, Diarrhoea, Modifiable risk factors, Children, Sub-Saharan Africa

## Abstract

**Background:**

Identifying the critical modifiable risk factors for acute respiratory tract infections (ARIs) and diarrhoea is crucial to reduce the burden of disease and mortality among children under 5 years of age in sub-Saharan Africa (SSA) and ultimately achieving the Sustainable Development Goals (SDGs). We investigated the modifiable risk factors of ARI and diarrhoea among children under five using nationally representative surveys.

**Methods:**

We used the most recent demographic and health survey (DHS) data (2014–2021) from 25 SSA countries, encompassing a total of 253,167 children. Countries were selected based on the availability of recent datasets (e.g., DHS-VII or DHS-VIII) that represent the current socioeconomic situations. Generalised linear latent mixed models were used to compute odds ratios (ORs). Population attributable fractions (PAFs) were calculated using adjusted ORs and prevalence estimates for key modifiable risk factors among ARI and diarrhoeal cases.

**Findings:**

This study involved 253,167 children, with a mean age of 28.7 (±17.3) months, and 50.5% were male. The highest PAFs for ARI were attributed to unclean cooking fuel (PAF = 15.7%; 95% CI: 8.1, 23.1), poor maternal education (PAF = 13.4%; 95% CI: 8.7, 18.5), delayed initiation of breastfeeding (PAF = 12.4%; 95% CI: 9.0, 15.3), and poor toilets (PAF = 8.5%; 95% CI: 4.7, 11.9). These four modifiable risk factors contributed to 41.5% (95% CI: 27.2, 52.9) of ARI cases in SSA. The largest PAFs of diarrhoea were observed for unclean cooking fuel (PAF = 17.3%; 95% CI: 13.5, 22.3), delayed initiation of breastfeeding (PAF = 9.2%; 95% CI: 7.5, 10.5), household poverty (PAF = 7.0%; 95% CI: 5.0, 9.1) and poor maternal education (PAF = 5.6%; 95% CI: 2.9, 8.8). These four modifiable risk factors contributed to 34.0% (95% CI: 26.2, 42.3) of cases of diarrhoea in SSA.

**Interpretation:**

This cross-sectional study identified four modifiable risk factors for ARI and diarrhoea that should be a priority for policymakers in SSA. Enhancing home-based care and leveraging female community health workers is crucial for accelerating the reduction in under-5 mortality linked to ARI and diarrhoea in SSA.

**Funding:**

None.

**Research in context**

**Evidence before this study**

We conducted a PubMed search on February 27, 2023, without language or date restrictions, using the search terms “acute respiratory tract infections” OR “diarrhoea” AND “population attributable fractions” OR “population attributable risk” OR “modifiable risk factors” AND “sub-Saharan Africa”. No studies were found that calculated the population attributable fraction (PAF) for key modifiable risk factors of acute respiratory tract infections (ARIs) and diarrhoea in SSA. While several studies have investigated different risk factors associated with ARI and diarrhoea in this region, they predominantly relied on relative measures of risk, such as odds ratios or relative risks (ORs/RRs). These measures may not directly contribute to public health planning and resource allocation. The public health impact of a strong association between a rare factor and an outcome may be limited, whereas a weaker association with a more prevalent factor could have a greater effect. We aimed to fill the knowledge gap.

**Added value of this study**

Our study adds value by utilising the PAF to examine the contribution of modifiable risk factors to the occurrence of ARI and diarrhoea in the study population of children aged under 5 years in SSA. PAF allows us to estimate the proportion of these diseases that can be attributed to these risk factors and the potential reduction in disease burden if the prevalence of these risk factors were hypothetically reduced to zero. Unlike ORs/RRs, PAF considers both the strength of the relationship between the risk factor and disease and the prevalence of the risk factor in the population. This comprehensive evaluation enables us to evaluate the overall public health impact and prioritise interventions that have the greatest potential for reducing the burden of ARI and diarrhoea more accurately.

**Implications of all the available evidence**

The global health community faces formidable challenges in meeting the health targets of the Sustainable Development Goals (SDGs) by 2030, which would require an estimated $371 billion in funding. These costs do not include the additional financial burdens associated with managing COVID-19 and the conflict in Ukraine. To eliminate preventable deaths among newborns and children in SSA caused by ARI and diarrhoea, it is crucial to invest in interventions that effectively utilise existing resources and capabilities. Our study underscored the importance of prioritising resources towards breastfeeding, maternal education, and improving household socioeconomic status as vital measures in reducing the burden of ARI and diarrhoea.

## Introduction

Diarrhoea and acute respiratory tract infections (ARIs) are the primary causes of morbidity and mortality in children under the age of five years worldwide.^1^, ^2^, ^3^ The United Nations (UN) Inter-agency Group for Child Mortality has revealed that together, these two conditions are responsible for a quarter of child deaths globally. Children experiencing diarrhoea and ARIs are at an increased risk of malnutrition and developmental issues.^4^^,^^5^ ARIs, such as pneumonia, can lead to long-term respiratory problems in children under-5.^6^ Diarrhoea and ARIs can also have substantial social and economic consequences, such as missed work or school and medical expenses.^7^ Children who experience multiple episodes of diarrhoea and ARIs may miss vital educational opportunities, impacting their future academic and economic prospects.^8^ Notably, diarrhoea and ARI are more prevalent in Low- and Middle-Income Countries, where access to quality healthcare services and sanitation facilities are limited.^2^^,^^3^

There are ongoing worldwide efforts to address the issue of diarrhoea and ARIs among children, as part of broader global health initiatives.^9^^,^^10^ The Sustainable Development Goals (SDG 3.2) aim to stop preventable deaths of newborns and children under-5 by focusing on reducing mortality rates from diarrhoea and ARI.^9^ The Integrated Global Action Plan for Pneumonia and Diarrhoea (GAPPD) has been developed to strengthen health systems, enhance access to vaccines and medications, and encourage better health practices to alleviate the burden of diarrhoea and ARI in children.^10^ Despite the global efforts to combat diarrhoea and ARI, these two conditions remain the leading causes of morbidity and mortality in Sub-Saharan Africa (SSA) and South Asia.^1^^,^^3^

Sub-Saharan Africa, despite its rich cultural heritage and history, the region faces various public health challenges. Children under-5 in SSA are particularly vulnerable, with diarrhoea being the second leading cause of death, accounting for about 16% of all deaths.^11^ Additionally, approximately half of all deaths related to pneumonia in children under-5 occur in the SSA region, while ARIs are responsible for roughly 10% of all deaths in this age group.^11^ Addressing the modifiable risk factors that contribute to diarrhoea and ARI in children is crucial for reducing the morbidity and mortality due to these illnesses.^10^

Past studies conducted in SSA countries have identified risk factors for ARI and diarrhoea, including low maternal education,^12^^,^^13^ poor households,^12^^,^^13^ suboptimal breastfeeding,^12^^,^^14^ distance from water sources,^12^ and lack of access to improved toilet facilities.^15^ However, previous studies analysing the impact of risk factors on outcomes have used relative measures of risks, such as odds ratios or relative risks (ORs/RRs), which might not directly help public health planning and resource allocation.^16^ For example, a strong association between a rare factor and an outcome may have limited public health significance, while a weaker association with a more prevalent factor may have a greater impact.

Using the Population Attributable Fraction (PAF) is a better approach because it considers both the strength of the relationship between the risk factor and disease and the prevalence of the risk factor in the population.^16^, ^17^, ^18^ This enables an estimate of the proportion of a specific outcome that can be potentially attributed to an exposure in the study population, as well as the potential reduction in the outcome if the exposure prevalence were hypothetically reduced to zero. It is important to acknowledge key assumptions of PAFs, including the independence of exposures and the unidirectional and constant associations between exposures and outcomes over time.^19^ Despite these assumptions, PAF estimates offer a concise method of quantifying risk and can complement other approaches in identifying modifiable risk factors for policy intervention and prioritisation.

In this study, we investigated the modifiable risk factors of diarrhoea and ARI among children under five using nationally representative surveys in 25 SSA countries. By identifying the critical modifiable risk factors for diarrhoea and ARI, targeted interventions can be implemented to reduce the burden of under-5 deaths attributable to these conditions in SSA and ultimately achieve the SDGs.^9^

## Methods

### Study design and data source

We used the most recent Demographic and Health Survey (DHS) dataset collected between 2014 and 2021, which encompassed 25 SSA countries. Countries were selected based on the availability of recent datasets (e.g., DHS-VII or DHS-VIII) that represent the current socioeconomic situations. Angola, Benin, Burundi, Cameroon, Chad, Ethiopia, Gambia, Ghana, Guinea, Kenya, Lesotho, Liberia, Madagascar, Malawi, Mali, Mauritania, Nigeria, Rwanda, Senegal, Sierra Leone, South Africa, Tanzania, Uganda, Zambia, and Zimbabwe were the countries included.

The DHS surveys are conducted by the health ministry or governmental agencies of each respective country, with support from the Inner-City Fund (ICF) International, at regular intervals. Standardised methods and instruments are used to ensure consistency in survey design and data collection. The DHS collects information on individuals' demographics and health, including topics such as maternal and child health, mortality, nutrition, and the social determinants of health.^20^

### Ethics

This study is a secondary analysis of publicly available anonymised data, and no ethical approval was required. The study followed the Strengthening the Reporting of Observational Studies in Epidemiology (STROBE) reporting guideline for cross-sectional studies.^21^

### Sampling procedure and sample size

The DHS surveys used a two-stage stratified cluster sampling design to select the study participants. The first stage involved grouping administrative units, such as States, as urban or rural strata, and randomly selecting Enumeration Areas (EAs) based on their size. In each selected EA, a complete census of households was conducted. In the second stage, a systematic random sampling technique was used to select a fixed number of households from each EA. For this study, the Child Dataset (Children’s Recode) was used, and dependent and independent variables were extracted for each country. The DHS survey data from 25 SSA countries were then combined and merged.^22^

The data were collected from eligible women, which included all females between the ages of 15 and 49 years who either lived in the households permanently or were present on the night before the survey.^22^ The weighted total of children across all 25 countries in the SSA region was 253,167.

### Outcome variables

The outcome variables were ARI and diarrhoea, measured based on the maternal recall of symptoms of cough and shortness of breath, and loose or liquid bowel movements, respectively.^22^ Diarrhoea was defined as having three or more loose or liquid bowel movements per day within the past two weeks.^22^ ARI was defined as the presence of cough along with rapid and shallow breathing within the past two weeks.^22^

### Modifiable risk factors

The modifiable risk factors were broadly categorised into three groups: child factors, maternal factors, and household factors. Child factors included perceived baby birth size, early initiation of breastfeeding, and duration of breastfeeding. The maternal factors included maternal body mass index (BMI), maternal education, maternal employment, antenatal care (ANC) visits, and place of birth. The household factors included household wealth index, type of toilet system, source of drinking water and type of cooking fuel (Table 1). Our selection of these modifiable risk factors was based on previously published studies in SSA,^23^^,^^24^ their statistical significance, data availability, and their potential for policy to improve child health and survival. Table 1 and Appendix 2 pp 2–5 present the definitions for variables.

**Table 1:** Definitions of modifiable risk factors for diarrhoea and acute respiratory tract infections among children under five in sub-Saharan Africa.

Modifiable risk factors	Definitions
**Child factors**	
Perceived birth size	Perceived birth size was grouped as ‘1’ = ‘Below average birth size’, or ‘2’ = ‘average and above birth size’.
Early initiation of breastfeeding (EIBF)	EIBF was grouped as ‘1’ = ‘initiated breastfeeding within 1 h of birth’, or ‘2’ = ‘Not initiated breastfeeding within 1 h of birth’
Duration of breastfeeding	Grouped as ‘1’ = ‘less than 12 months’ or ‘2’ = ‘12 months or more’
**Maternal factors**	
Maternal body mass index (BMI)	BMI was calculated as a woman’s weight in kilograms divided by the square of her height in meters (kg/m^2^). For this study, BMI was grouped as ‘1’ = ‘underweight (BMI < 18.5 kg/m^2^)’, ‘2’ = ‘normal weight (BMI ≥ 18.5 kg/m^2^ and BMI ≤ 24.9 kg/m^2^)’, or ‘3’ = ‘overweight or obese (BMI ≥ 25.0 kg/m^2^)’
Maternal education	Maternal education was grouped as ‘1’ = ‘no schooling’, ‘2’ = ‘primary education’, or ‘3’ = ‘secondary education or higher’.
Maternal employment	Maternal employment status was grouped as “not working” or “working”
Frequency of antenatal care (ANC) visits	Frequency of ANC visits was grouped as ‘1’ = ‘none’, ‘2’ = ‘1–4 visits’, or ‘3’ = ‘four and above visits’
Place of birth	Place of birth was grouped as ‘home ‘or ‘health facility birth’
**Household factors**	
Household wealth index	Household wealth index was grouped as ‘1’ = ‘poor’, ‘2’ = ‘middle’, or ‘3’ = ‘rich’
Source of drinking water and type of toilet facility	The source of drinking water and type of toilet facility were grouped as ‘1’ = ‘improved’ or ‘2’ = ‘not improved’
Type of cooking fuel	Type of cooking fuel was grouped as ‘1’ = ‘cleaned’ or ‘2’ = ‘not cleaned’

### Potential covariates

For this study, we considered potential covariates including sex of the baby (grouped as male or female), birth order (grouped as first child, two up to five births or five or above births), maternal age (grouped as 15–24 years, 25–34 years, or 35–49 years) and place of residence (grouped as rural or urban). Consistent with principles of the disjunctive cause criterion,^25^^,^^26^ our choice of covariates adhered to three criteria: (i) existing literature,^23^^,^^24^ (ii) exclusion of variables that might serve as potential mediators (those with a plausible causal link between modifiable risk factors and outcomes), and (iii) their correlation with the outcome.

### Statistical analysis

To examine the modifiable risk factors, a three-stage analytical strategy was used. In the initial stage, we calculated frequencies and percentages to provide an overview of the study population and the prevalence of ARI and diarrhoea across the study factors. In the second stage, we fit the Generalised Linear Latent and Mixed Models (GLLAMM) to determine the odds ratios (ORs) and 95% confidence intervals (CIs) for modifiable risk factors of ARI and diarrhoea, based on previously published studies.^23^^,^^24^^,^^27^

The GLLAMM models were constructed in four steps. Initially, a null unconditional model was developed in step one, without any study variable. In step two, individual-level factors (including child, maternal, and household factors) were incorporated into the model. Step three introduced community-level factors (specifically, place of residence) without variables from step two. The final model encompassed both individual and community-level factors. This final model, which included both individual and community-level factors, was chosen due to its lower deviance and better ability to explain the variation in the outcome variables (Appendix 3 pp 5–7). Additionally, Appendix 3 pp 8–9 are presented to show the assumptions of the GLLAMM model, including the normal distribution of random effects and scatter plots of residuals against fitted values for ARI and diarrhoea.

In the last stage, once the modifiable risk factors for diarrhoea and ARI were identified in the GLLAMM analysis, we calculated PAFs using Miettinen’s formula. The choice of Miettinen's formula was based on its ability to provide valid estimates even in the presence of confounding, particularly when using adjusted ORs.^28^ PAF quantifies the percentage of diarrhoea and ARI cases in SSA that could potentially be averted by addressing the identified modifiable risk factors among the populations.^29^

PAF was calculated using the following formula:

PAF=Pc(OR−1)/OR

where Pc is the prevalence of the modifiable risk factor among cases, and OR is the adjusted odds ratios of diarrhoea and ARI associated with the modifiable risk factors.^28^^,^^29^ Because risk factors tend to occur together within individuals, adding up the PAFs of each risk factor would result in an inflated estimate of their combined PAFs. Thus, we calculated a joint PAF across all risk factors using formula below^30^^,^^31^:

$$PAF\;\left(combined\right)=1-\prod_{r=1}^{R}1\;-\;\text{PAFr}$$

where *r* represents each modifiable risk factor.

We used 'svy' command in STATA (version 15.0, Stata Corp, College Station, TX, USA) to account for sampling weights addressing unequal probabilities in household selections and non-responses, as well as to account for clustering and stratification.^32^ In addition, denormalisation of the sampling weight was applied to the combined dataset, and a new weight at the population level was created to consider the unequal distribution of population across SSA countries. This step was crucial because the size of the population in some countries (e.g., Nigeria) was significantly greater than others (e.g., Gambia), which had the potential to bias the results. The regression modelling was conducted using the ‘GLLAMM’ package for STATA.^33^ The measure of association between modifiable risk factors and the outcome variables was reported as ORs, PAFs, and 95% confidence intervals.

### Role of the funding source

No funding was received for this work.

## Results

This study included 253,167 children from 25 SSA countries. The children had a mean age of 28.7 (±17.3) months, and 50.5% were males. A total of 151,287 (59.8%) children did not receive breastmilk within the first hour of birth, and 183,667 (72.5%) mothers had no or a primary level of education. A total of 101,400 (56.8%) mothers had four or more ANC visits and two-thirds 160,507 (63.4%) were delivered at a health facility. A total of 129,880 (52.7%) children resided in households with an unimproved toilet, and the majority 219,268 (86.7) of households used unclean cooking fuel [Table 2]. Additional ARI and diarrhoea prevalence data is available in Appendices 5 and 6.

**Table 2:** Characteristics of study participants among children under five in sub-Saharan Africa countries, 2014–2021 (N = 253,166).

Variables	Male	Female	Total population
	n (%)	n (%)	n (%)
**Child factors**			
Perceived baby birth size			
Below average	18,476 (15.0)	22,822 (18.9)	41,298 (16.9)
Average or above average	104,671 (85.0)	97,753 (81.1)	202,424 (83.1)
Early initiation of breastfeeding			
No	76,786 (60.0)	74,501 (59.5)	151,287 (59.8)
Yes	51,176 (40.0)	50,703 (40.50)	101,880 (40.2)
Duration of breastfeeding			
≤12 months	28,996 (23.6)	28,174 (23.4)	57,170 (23.5)
>12 months	93,893 (76.4)	92,176 (76.6)	186,069 (76.5)
**Maternal factors**			
Maternal age			
15–24 years	35,808 (28.0)	34,728 (27.7)	70,536 (27.9)
25–34 years	61,959 (48.4)	60,621 (48.4)	122,581 (48.4)
35+ years	30,195 (23.6)	29,855 (23.8)	60,050 (23.7)
Birth order			
One	27,389 (21.4)	26,868 (21.5)	54,257 (21.4)
2–4 children	62,345 (48.7)	60,687 (48.5)	123,032 (48.6)
5+ children	38,228 (29.9)	37,649 (30.1)	75,877 (30.0)
Maternal education			
No or low education	92,493 (72.3)	91,174 (72.8)	183,667 (72.5)
Secondary or higher	35,467 (27.7)	34,028 (27.2)	69,495 (27.5)
Maternal employment			
Not working	34,063 (41.9)	33,321 (42.1)	67,384 (42.0)
Working	47,279 (58.1)	45,810 (57.9)	93,089 (58.0)
Antenatal care			
Three or less visits	39,066 (43.2)	38,072 (43.2)	77,138 (43.2)
4+ visits	51,420 (56.8)	49,981 (56.8)	101,400 (56.8)
Place of birth			
Home	46,192 (36.1)	46,349 (37.0)	92,541 (36.6)
Health facility	81,706 (63.9)	78,801 (63.0)	160,507 (63.4)
**Household factors**			
Household wealth			
Poor	57,262 (44.7)	56,187 (44.9)	113,448 (44.8)
Middle	25,661 (20.1)	24,988 (20.0)	50,649 (20.0)
Rich	45,040 (35.2)	44,030 (35.2)	89,069 (35.2)
Type of toilet system			
Not improved	65,687 (52.7)	64,193 (52.6)	129,880 (52.7)
Improved	59,005 (47.3)	57,751 (47.4)	116,755 (47.3)
Source of drinking water			
Not protected	57,665 (45.1)	56,856 (45.4)	114,521 (45.2)
Protected	70,297 (54.9)	68,348 (54.6)	138,646 (54.8)
Type of cooking fuel			
Not cleaned	110,791 (86.7)	16,540 (13.2)	219,268 (86.7)
Cleaned	16,993 (13.3)	108,478 (86.8)	33,533 (13.3)
**Community level factors**			
Place of residence			
Urban	39,952 (31.2)	39,041 (31.2)	78,992 (31.2)
Rural	88,011 (68.8)	86,164 (68.8)	174,175 (68.8)

**Fig. 1:**
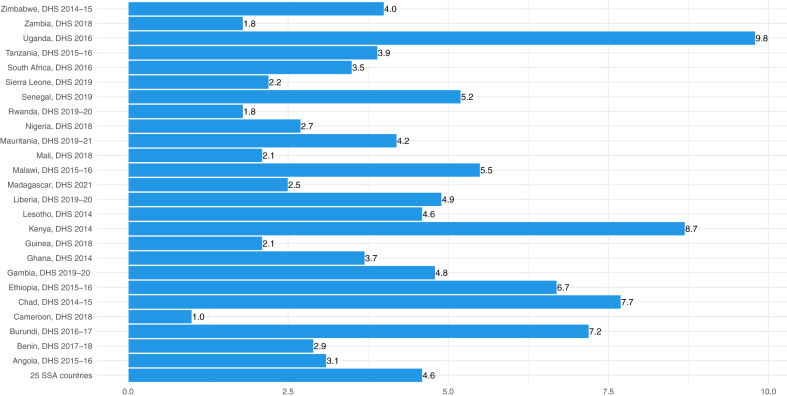
Prevalence of acute respiratory tract infections among children under-5 in sub-Saharan Africa countries. i) DHS represents demographic and health survey; ii) SSA represents sub-Saharan Africa.

**Fig. 2:**
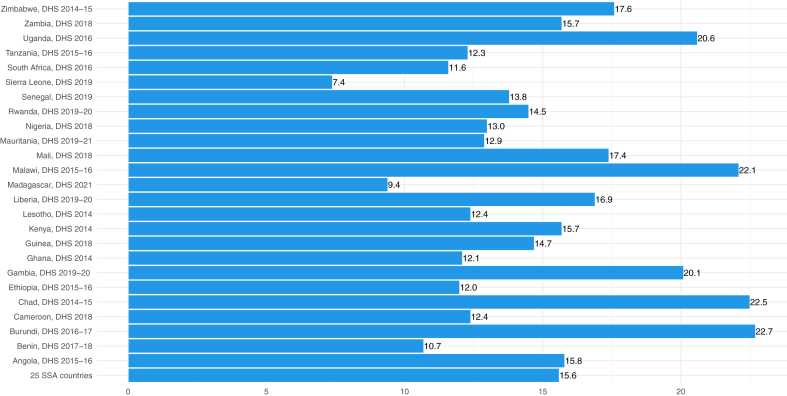
Prevalence of diarrhoea among children under-5 in sub-Saharan Africa countries. i) DHS represents demographic and health survey; ii) SSA represents sub-Saharan Africa.

Figs. 1 and 2 presented the prevalence of ARI and diarrhoea across 25 SSA countries. The overall prevalence of ARI among the 25 SSA countries was 4.6% with a 95% CI from 4.5% to 4.8%. The highest prevalence was observed in Uganda (Prevalence = 9.8%; 95% CI: 9.1%, 10.5%), followed by Kenya (Prevalence = 8.7%; 95% CI: 8.1%, 9.2%), while the lowest prevalence was observed in Cameroon (Prevalence = 1.0%; 95% CI: 0.8%, 1.3%) [Fig. 1 and Appendix 4 p 10].

The overall prevalence of diarrhoea in 25 SSA was 15.6% (95% CI from 15.3% to 15.8%). The highest prevalence of diarrhoea was in Burundi (Prevalence = 22.7%; 95% CI: 21.6%, 23.8%), followed by Chad (Prevalence = 22.5%; 95% CI: 21.2%, 23.8%), while the lowest prevalence was in Sierra Leone (Prevalence = 7.4%; 95% CI: 6.6%, 8.3%) [Fig. 2 and Appendix 4 p 10].

The highest proportion PAFs of ARI were attributed to unclean cooking fuel (PAF = 15.7%; 95% CI: 8.1, 23.1), poor maternal education (PAF = 13.4%; 95% CI: 8.7, 18.5), delayed initiation of breastfeeding after birth (PAF = 12.4%; 95% CI: 9.0, 15.3), poor household toilets (PAF = 8.5%; 95% CI: 4.7, 11.9) and household poverty (PAF = 3.6%; 95% CI: 0.5, 7.3) [Table 3]. The combined PAFs showed that 41.5% (95% CI: 27.2, 52.9) cases of ARI in SSA were attributed to unclean cooking fuel, poor maternal education, delayed initiation of breastfeeding after birth, and poor household toilets.

**Table 3:** Determinants and population attributable fractions for acute respiratory tract infections in sub-Saharan African countries.

Variables	Proportion of exposure cases (95% CI)^a^	OR (95% CI)^b^	PAF % (95% CI)
**Child factors**			
Perceived baby birth size			
Below average	20.6 (19.6, 21.7)	1.26 (1.17, 1.35)	4.25 (2.85, 5.63)
Average or above average	79.4 (78.4, 80.5)	Ref	Ref
Early initiation of breastfeeding			
No	60.3 (59.0, 61.7)	1.26 (1.18, 1.33)	12.44 (9.00, 15.31)
Yes	39.7 (38.3, 41.0)	Ref	Ref
Duration of breastfeeding			
≤12 months	27.8 (26.8, 28.9)	1.09 (1.03, 1.16)	2.30 (0.78, 4.00)
>12 months	72.2 (71.1, 73.2)	Ref	Ref
**Maternal factors**			
Maternal education			
No or low education	77.1 (75.9, 78.3)	1.21 (1.13, 1.31)	13.38 (8.73, 18.53)
Secondary or higher	22.9 (21.7, 24.1)	Ref	Ref
Maternal employment			
Not working	42.1 (40.1, 44.1)	1.08 (1.02, 1.15)	3.12 (0.79, 5.75)
Working	57.9 (55.9, 59.9)	Ref	Ref
Antenatal care			
Three or less visits	45.2 (43.8, 46.7)	0.96 (0.90, 1.02)	−1.89 (−4.87, 0.92)^c^
4 or more visits	54.8 (53.4, 56.2)	Ref	Ref
Place of birth			
Home	39.4 (37.8, 41.0)	1.06 (0.99, 1.13)	2.23 (−0.38, 5.96)
Health facility	60.6 (59.0, 62.2)	Ref	Ref
**Household factors**			
Household wealth			
Poor	48.8 (47.3, 50.3)	1.08 (1.00, 1.17)	3.61 (0.00, 7.31)
Middle	20.3 (19.2, 21.4)	1.02 (0.94, 1.11)	0.40 (−1.23, 2.12)
Rich	30.9 (29.5, 32.4)	Ref	Ref
Type of toilet system			
Not improved	58.8 (56.3, 59.6)	1.17 (1.09, 1.25)	8.54 (4.65, 11.92)
Improved	42.0 (40.4, 43.7)	Ref	Ref
Type of cooking fuel			
Not cleaned	90.3 (89.3, 91.2)	1.21 (1.10, 1.34)	15.67 (8.12, 23.14)
Cleaned	9.7 (8.8, 10.7)	Ref	Ref

a Weighted count and proportion for acute respiratory tract infections.

b Odds ratio (OR) estimates for each are adjusted for all other modifiable risk factors and place of residence; Population attributable fractions (PAF).

c Negative PAF indicates the Prevented Fraction (PF), which is the proportion of outcome that could be avoided if it were possible to expose everyone to this protective factor. However, all negative PAFs for ARI were not statistically significant.

The largest PAFs of diarrhoea were observed for unclean cooking fuel (PAF = 17.3%; 95% CI: 13.5, 22.3), delayed initiation of breastfeeding (PAF = 9.2%; 95% CI: 7.5, 10.5), poor households (PAF = 7.0%; 95% CI: 5.0, 9.1) and poor maternal education (PAF = 5.6%; 95% CI: 2.9, 8.8) [Table 4]. The combined PAFs showed that 34.0% (95% CI: 26.2, 42.3) cases of diarrhoea in SSA were attributed to unclean cooking fuel, delayed initiation of breastfeeding after birth, poor households, and poor maternal education.

**Table 4:** Determinants and population attributable fractions for diarrhoea in sub-Saharan African countries.

Variables	Proportion of exposure (95% CI)^a^	OR (95% CI)^b^	PAF % (95% CI)
**Child factors**			
Perceived baby birth size			
Below average	19.7 (19.1, 20.3)	1.18 (1.14, 1.23)	3.01 (2.35, 3.80)
Average or above average	80.3 (79.7.1, 80.9)	Ref	Ref
Early initiation of breastfeeding			
No	55.2 (54.4, 56.1)	1.20 (1.16, 1.23)	9.20 (7.50, 10.49)
Yes	44.8 (43.9, 45.6)	Ref	Ref
Duration of breastfeeding			
≤12 months	28.4 (27.9, 29.0)	1.02 (0.99, 1.06)	0.55 (−0.28, 1.64)
>12 months	71.6 (71.0, 72.1)	Ref	Ref
**Maternal factors**			
Maternal education			
No or low education	75.4 (74.7, 76.2)	1.08 (1.04, 1.13)	5.59 (2.87, 8.77)
Secondary or higher	24.6 (23.8, 25.3)	Ref	Ref
Maternal employment			
Not working	41.6 (40.6, 42.7)	1.04 (1.00, 1.07)	1.60 (0.00, 2.79)
Working	58.4 (57.3, 59.4)	Ref	Ref
Antenatal care			
Three or less visits	44.7 (43.9, 45.5)	0.98 (0.95, 1.01)	−0.91 (−2.31, 0.45)^c^
4 or more visits	55.3 (54.5, 56.1)	Ref	Ref
Place of birth			
Home	36.3 (35.3, 37.3)	0.98 (0.95, 1.00)	−0.74 (−1.86, 0.00)^c^
Health facility	63.7 (62.7, 64.7)	Ref	Ref
**Household factors**			
Household wealth			
Poor	47.9 (46.8, 48.9)	1.17 (1.12, 1.23)	6.96 (5.01, 9.14)
Middle	20.0 (19.4, 20.6)	1.13 (1.08, 1.18)	2.30 (1.44, 3.14)
Rich	32.1 (31.1, 33.2)	Ref	Ref
Type of toilet system			
Not improved	54.5 (53.4, 55.5)	1.03 (0.99, 1.07)	1.59 (−0.54, 3.63)^c^
Improved	45.5 (44.5, 46.6)	Ref	Ref
Source of drinking water			
Not protected	44.6 (43.5, 45.7)	0.97 (0.94, 1.01)	−1.38 (−2.78, 0.46)^c^
Protected	55.4 (54.3, 56.5)	Ref	Ref
Type of cooking fuel			
Not cleaned	89.1 (88.4, 89.8)	1.24 (1.18, 1.33)	17.25 (13.48, 22.28)
Cleaned	10.9 (10.2, 11.6)	Ref	Ref

a Weighted count and proportion for diarrhoea.

b Odds ratio (OR) estimates for each are adjusted for all other modifiable risk factors and place of residence; Population attributable fractions (PAF).

c Negative PAF indicates the Prevented Fraction (PF), which is the proportion of outcome that could be avoided if it were possible to expose everyone to this protective factor. The potential explanation for unexpected finding related to place of birth could be that those mothers who visited health facilities for delivery may be associated with complicated delivery, given the low level of institutional delivery in most sub-Saharan African countries.

## Discussion

To the best of our knowledge, this is the first study to compute PAFs for key modifiable risk factors of ARIs and diarrhoea using nationally representative surveys in SSA. Our findings revealed that the highest PAFs of ARI were attributed to unclean cooking fuel, poor maternal education, delayed initiation of breastfeeding, and unimproved household toilets. These four modifiable risk factors contributed to 41.5% of cases of ARI in SSA. The largest PAFs of diarrhoea were observed for unclean cooking fuel, delayed initiation of breastfeeding, household poverty, and poor maternal education. These four modifiable risk factors contributed to 34.0% of cases of diarrhoea in SSA.

The global health community is currently confronted with formidable obstacles in fulfilling the SDG health targets by 2030, which will require an estimated $371 billion in costs, not including the additional costs associated with managing COVID-19 and the health implications of the conflict in Ukraine.^34^ Ending preventable deaths of newborns and children attributable to ARI and diarrhoea requires investments that are directed toward the most effective interventions that maximise existing resources and capabilities. This study showed the significance of prioritising resources for breastfeeding, maternal education and improving household socioeconomic status. Our results can guide the allocation of funding, inform public health strategies, and shape policy priorities aimed at tackling ARI and diarrhoea.

The World Health Organization and the United Nations Children’s Fund (WHO/UNICEF) recommend optimal breastfeeding until the child is two years old, considering the immunological, nutritional, hygienic, economic, and psychological benefits of breastmilk for infants, mothers, and communities.^35^ This study showed the importance of early breastfeeding initiation in the first hour of birth as a key priority to reduce child morbidity related to ARI and diarrhea in SSA. The Baby Friendly Hospital Initiative (BFHI) effectively promotes early initiation of breastfeeding in health facilities.^36^ Nevertheless, a recent systematic review has highlighted substantial challenges in health facility infrastructure, supplies, and staffing for the initiation and implementation of BFHI in SSA.^37^ Our findings suggest the need for additional resources to address the barriers for the successful implementation of BFHI practices in SSA healthcare facilities.

Improvements in maternal education at the population level are undeniably one of the most pivotal and well-researched factors contributing to the improvement of child health and survival.^38^ Notably, adapting education for adult women in Africa is not only feasible but essential. It is crucial therefore to recognise the potential role of community-based female healthcare workers in educating female heads of households about essential aspects such as proper diet during pregnancy, breastfeeding, the benefits of healthcare deliveries, culturally suitable weaning foods, microenterprise, and clean household environment for their growing families.

The calculation of PAFs for ARIs and diarrhoea presents an opportunity to guide the allocation of resources to reduce the number of deaths among newborns and children attributed to these conditions in SSA. Furthermore, by utilising nationally representative datasets from the DHS in our study, the generalisability of our findings to the region is enhanced. The modifiable risk factors investigated in this study also hold broader implications for child health policies and have the potential to impact other early-life and child health outcomes.

This study also had limitations. Firstly, the use of cross-sectional data makes it difficult for the establishment of a clear temporal relationship between certain potential modifiable risk factors and the outcome. Secondly, recall bias may have affected results for certain modifiable risk factors, like dairy product consumption, early initiation of breastfeeding and duration of breastfeeding. However, we minimised this bias by focusing on the youngest child in the study population. Thirdly, misclassification bias, including the misclassification of common colds as ARIs or minor changes in bowel habits as diarrhea, as well as potential errors in categorising household characteristics such as cooking fuel and sanitation facilities, may have influenced the results.

PAF estimates rely on assumptions of causal relationships, independence of modifiable risk factors, and consistent associations over time.^19^ However, these assumptions may be unlikely given the complex interactions between socio-economic, cultural, health service, maternal, and child factors associated with ARI and diarrhoea. Additionally, the joint PAF calculation assumes no association and interaction between modifiable risk factors, both of which are unrealistic in the present study setting. Despite these limitations, PAFs offer a straightforward and intuitive metric that can complement other approaches in identifying modifiable risk factors for policy intervention when prioritising interventions.

Potential bias in prevalence data used is discussed above, but unmeasured confounders (e.g., household food security, socio-cultural factors) may persist, potentially leading to over- or underestimation of the PAF. Nevertheless, we examined household socioeconomic status as a proxy for food security and socio-cultural practices. Lastly, assessing individual modifiable risk factors assumes other factors remain constant, which may not always be the case. Multivariable adjustment of risk factors and covariates may help minimise potential violations of this assumption to some degree.

In conclusion, this cross-sectional study identified four modifiable risk factors that were responsible for 41.5% of ARI and 34.0% of diarrhoea cases in SSA. Given the current state of the global economy, we suggest that policymakers prioritise these factors when designing child health interventions that aim to combat ARI and diarrhoea. The involvement of female healthcare workers will be crucial in the implementation of such interventions, particularly in rural Africa, where they will need to reach female heads-of-households.

## Contributors

KYA conceived the idea, downloaded the data, did data management, conducted the statistical analyses, and drafted the manuscript. AGR and AFD conducted the literature review and helped draft the manuscript. KYA wrote the first draft of the manuscript, which was critically revised by all authors. AFD and GDK accessed and verified the data, and all authors had the final responsibility of whether to submit the manuscript or not.

## Data sharing statement

All DHS data are available at https://dhsprogram.com/data/available-datasets.cfm. The DHS provides open access to survey data files for legitimate academic research purposes. To initiate the download process, registration is mandatory. Researchers are required to provide their contact information, research title, and a brief description of the proposed analysis. Approval for dataset access is typically confirmed via email. It is important to note that these datasets are third-party resources and not under the ownership or collection of the authors, who possess no special access privileges. Analysis files created from this data can be requested from the corresponding author. Appendix 7 p 13 provides the data access grant letter for this project.

## Declaration of interests

All other authors declare no competing interests.
