# Supplementary material for: Population modifiable risk factors associated with under-5 acute respiratory tract infections and diarrhoea in 25 countries in sub-Saharan Africa (2014–2021): an analysis of data from demographic and health surveys
Source: eClinicalMedicine. 2024 Feb 3;68:102444. doi: 10.1016/j.eclinm.2024.102444 (PMC10850409; doi:10.1016/j.eclinm.2024.102444)
Supplement: Appendix [file mmc1.pdf]

Appendix 1: List of countries and sample size included

List of countries	Weighted sample	Response rates for women's questionnaire
Angola	13180	94.2%
Benin	12146	97.6%
Burundi	12331	97.6%
Cameroon	8711	98.0%
Chad	16387	92.1%
Ethiopia	10062	94.6%
The Gambia	6913	95.1%
Ghana	5233	97.3%
Guinea	6746	99.0%
Kenya	17896	96.6%
Lesotho	2652	97.1%
Liberia	4255	96.4%
Madagascar	11112	94.9%
Malawi	15840	97.7%
Mali	9248	97.6%
Mauritania	10581	96.2%
Nigeria	29486	99.3%
Rwanda	7717	99.7%
Senegal	5205	96.1%
Sierra Leone	7998	96.7%
South Africa	2849	86.2%
Tanzania	8835	97.3%
Uganda	13320	97.0%
Zambia	9071	96.4%
Zimbabwe	5393	96.2%

Appendix 2: Variables measurement

Modifiable risk factors

The modifiable risk factors were broadly categorized into three groups: child factors (perceived baby birth size, early initiation of breastfeeding, and duration of breastfeeding), maternal factors (maternal body mass index, maternal education, maternal employment, ANC visits, and place of birth.) and household factors (household wealth index, type of toilet system, source of drinking water, and type of cooking fuel). This classification is based on previously published studies in SSA[1-4].

Perceived birth size

The size of the child at birth is classified as small or very small, and average or larger, and is based on the mother's report of the relative size of the child at birth. For this study, we grouped small or very small as "*below average*" and average or larger as "*average and above birth size*", based on previously published study[5].

Early initiation of breastfeeding

The initiation of breastfeeding indicators was reported for all children born in the 5 years before the survey. We calculated early initiation of breastfeeding (EIBF) as a percentage of children who started breastfeeding within one hour of birth. For this study, EIBF was grouped as '1' = '*initiated breastfeeding within 1 h of birth*', or '2' = '*Not initiated breastfeeding within 1 h of birth*', based on the previously published studies[5,6].

Duration of breastfeeding

In DHS, the median duration of breastfeeding is reported. For this study, we grouped the duration of breastfeeding as '1' = '*less than 12 months*' or '2' = *'12 months or more*'[7].

Maternal BMI

Maternal nutritional status was measured using the WHO adult BMI classification[8]. In DHS, weight measurements were obtained using lightweight SECA mother-infant scales with a digital screen designed and manufactured under the guidance of UNICEF. Height measurements were carried out using a Shorr measuring board. The BMI is calculated as weight in kilograms divided by the square of height in meters. For this study, BMI was grouped as '1' = '*underweight (BMI < 18.5 kg/m2)*', '2' = '*normal weight (BMI ≥ 18.5 kg/m2 and BMI ≤ 24.9 kg/m2)*', or '3' = '*overweight or obese (BMI ≥ 25.0 kg/m2)*', based on previously published studies[9,10].

Maternal education

In DHS, maternal education is generally reported as the highest level of education attended (not necessarily completed) (in categories of no education, primary, secondary, higher than secondary. For this study, we regrouped maternal education as '1' = '*no schooling*', '2' = '*primary education*', or '3' = '*secondary education or higher*'.

Maternal occupation

Maternal occupation was categorized into two groups. Mothers working in formal (such as professional, technical, managerial, clerical, and services areas) and informal sectors (those who were working in agricultural and manual works) were grouped as "*working*" and non-working mothers were grouped as "*Not working*".

ANC visits and place of birth

The number of antenatal visits is grouped into categories of no antenatal care, 1-3 visits, and 4+ visits, and place of birth was grouped as *home* or *health facility*.

Wealth Index

The wealth index represents a combined measurement of a household's overall living standards. It is determined by assessing various factors, including the household's ownership of specific assets like

televisions and cars, dwelling features such as flooring material, drinking water source, and toilet facilities. Each asset's importance is calculated using factor scores derived from principal components analysis (PCA). These scores are standardized to have a mean of zero and a standard deviation of one. Based on whether a household owns a particular asset, standardized scores are assigned and summed up. Individuals are then ranked according to their household's total score. Detailed procedures for wealth index construction are available elsewhere[11].

The population is divided into five equal groups, or quintiles, to establish wealth categories: Lowest, Second, Middle, Fourth, and Highest. For this study, the household wealth index was regrouped as '1' = 'poor (lowest and second quintile)', '2' = 'middle', or '3' = 'rich (fourth and highest quintile)', based on previously published studies[5-7,9,10].

Type of cooking fuel

The study also considered the type of cooking fuel among modifiable risk factors. For this study, households that used electricity, natural gas, biogas, or kerosene as a cooking fuel were classified as '*clean'*, while those households that used charcoal, firewood, or dung were grouped as '*not clean'*. This classification is based on previously published studies[12,13].

Source of drinking water

The source of drinking water and type of toilet facility were classified as 'improved' or 'not improved', based on the taxonomy of the WHO and UNICEF Joint Monitoring Programme (JMP) for Water and Sanitation[14] as applied in past studies[4,6]. Households that used piped water, public tap or standpipe, a tube well or borehole, a protected well/spring, rainwater and/or bottled water were classified as '*improved'*. Households that used unprotected well/spring, tanker truck/cart, surface water, and/or sachet water were grouped as '*not improved*'[14].

Type of toilet system

The type of toilet facility was also grouped as '*improved'* (included flush/pour-flush toilets or flush/pour-flush toilets piped to the sewer system, septic tank or pit latrine; ventilated improved pit (VIP) latrine; pit latrine with slab and/or composting toilet). '*Not improved*' type of facility included flush/pour-flush not piped to sewer, septic tank or pit latrine; pit latrine without slab/open pit; bucket or hanging toilet/hanging latrine and no facility/bush/field[14].

Appendix 3: Statistical analysis

Step 1: descriptive statistics

Frequencies and percentages were calculated to provide an overview of the study population and the prevalence of ARI and diarrhoea across the study factors. All descriptive analyses accounted for the sampling weights, clustering, and stratification using the 'svy' command in STATA. Appendix 4 and 5 presented to show the prevalence of ARI and diarrhoea across the study factors.

Step 2: Generalised linear latent and mixed models

Variable selection

The modifiable factors were selected based on past literature[1-4] their importance for the outcomes, availability of data, and the amenability for policy interventions in improving child health and survival. In this study, maternal BMI was excluded due to the missing maternal BMI for some countries (e.g., Angola).

Our selection of covariates was based on: 1) previously published studies[3,4], (ii) by excluding potential mediators (variables with a potential causal link between modifiable risk factors and outcomes), and (iii) their statistical significance with the outcome. In our analysis, we initially considered the gender of the baby, birth order, maternal age, and place of residence as potential covariates, as they were less likely to be part of the causal pathway. However, in the final model, only the place of residence retained the significant associations with the outcome.

The Generalised Linear Latent and Mixed Models (GLLAMM) were used to determine the odds ratios (ORs) and 95% confidence intervals for modifiable risk factors of ARI and diarrhoea.

Our GLLAMM models were structured in two levels, individual (e.g., child, maternal and household factors) and community levels (place of residence) to account for the hierarchical nature of the data, wherein children under five years old are nested within geographic clusters. The random effects and model fitness outputs are presented in the below table.

Multilevel modelling offers distinct advantages compared to classical single-level logistic regression models. Firstly, it acknowledges the hierarchical nature of data, recognizing that children under-five (level I) is nested within clusters (level II). Failure to account for these hierarchies results in underestimated standard errors of regression coefficients, leading to an overstatement of statistical significance. Secondly, multilevel modelling addresses the dependence of observations within the same clusters; children within the same cluster tend to be more similar than those in different clusters. Lastly, it allows for the simultaneous estimation of cluster-level effects (random effects) and the assessment of associations for community-level predictors, such as place of residence.

The multilevel models were constructed in four steps. Initially, a null unconditional model was developed in stage one, without any study variable. In stage two, individual-level factors (including child, maternal, and household factors) were incorporated into the model. Stage three introduced community-level factors (specifically, place of residence) without variables from stage two. The ultimate model, presented in the results, encompassed both individual and community-level factors. This final model, which included both individual and community-level factors, was chosen due to its minimal deviance and superior ability to explain the variation in the outcome variables. The table below displays the random variation and model fitness test results for the fitted models.

Random effects and model fitness:

	Model I	Model II	Model III	Model IV
ARI				
Random effects				
ICC	0.86	0.86	0.87	0.85
Model fitness				
Log-likelihood	41020.2	18117.9	41176.9	18123.3
AIC	-82034.6	-36205.7	-82345.8	-36214.6
BIC	-82003.2	-36061.5	-82304	-36060.8
Diarrhoea				
Random effects				
ICC	0.19	0.18	0.19	0.18
Model fitness				
Log-likelihood	-100034.9	-49286.9	-99970.6	-49286.3
AIC	200075.9	98603.9	199949.2	98604.6
BIC	200107.2	98748.1	199991	98758.5

Step 3: Population-attributable fractions

PAF quantifies the percentage of diarrhoea and ARI cases in SSA that could potentially be averted by addressing the identified modifiable risk factors among the populations[15]. Once the modifiable risk factors for diarrhoea and ARI were identified in the GLLAMM analysis, we calculated the PAFs using Miettinen’s formula (formula 1). The choice of Miettinen's formula was based on its ability to provide valid estimates even in the presence of confounding, particularly when using adjusted ORs[16,17].

$$\boldsymbol{PAF = Pc(OR-1)/\,OR}\text{********************************}\textbf{(1)}$$

Where Pc is the prevalence of the modifiable risk factor among cases, and OR is the adjusted odds ratio of diarrhoea and ARI associated with the modifiable risk factors. Because risk factors tend to occur together within individuals, adding up the PAFs of each risk factor would result in an inflated estimate of their combined PAFs. Thus, we calculated a joint PAF across all risk factors using formula 2 [3,18]:

$$\boldsymbol{PAF\ (combined) = 1 - \prod_{r=1}^{R}(1 - PAFr)}\quad \text{*************}\textbf{\textit{(2)}}$$

Where *r* represents each modifiable risk factor.

Step 4: Checking assumptions

We checked our model estimation for the normal distribution of Random Effects and scatter plot of residuals against fitted values.

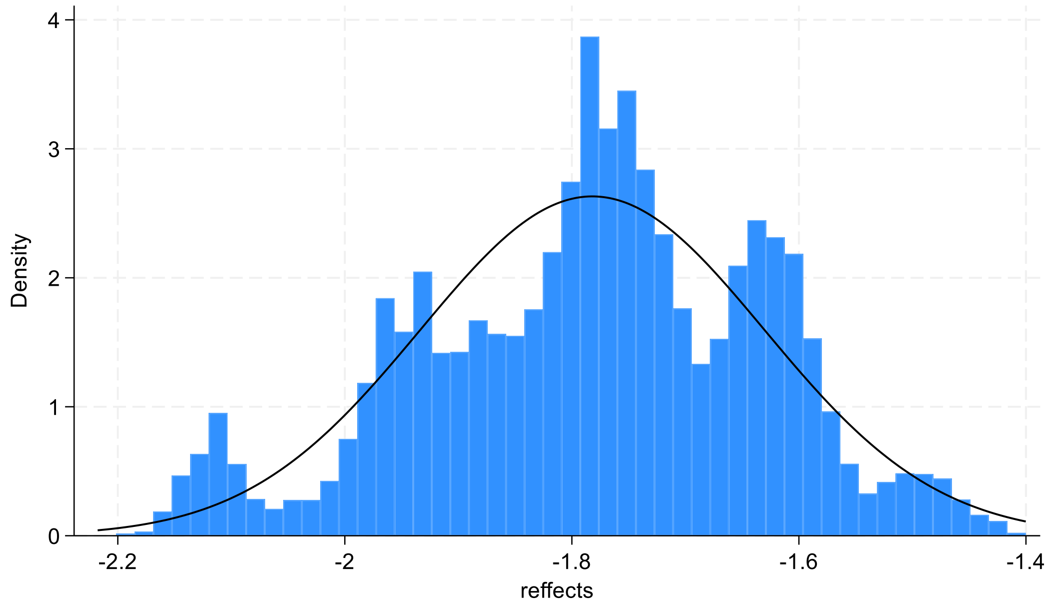

Figure 1: Histogram showing the normal distribution of random effects for diarrhoea.

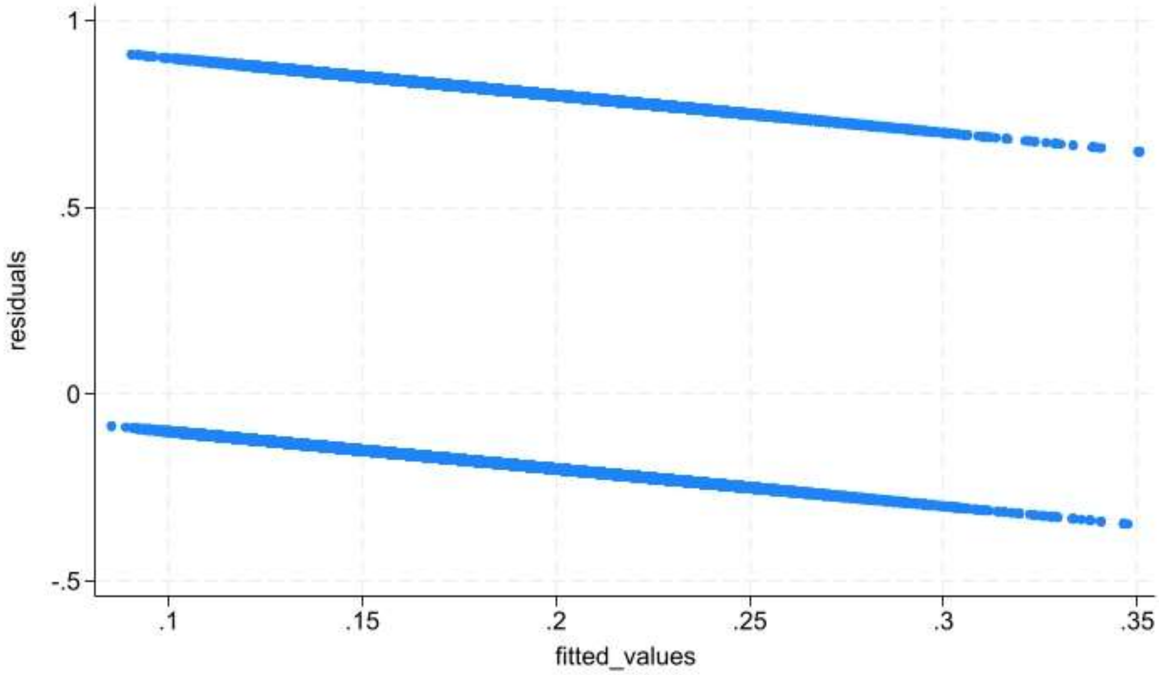

Figure 2: Scatter plot of residuals against fitted values for diarrhoea.

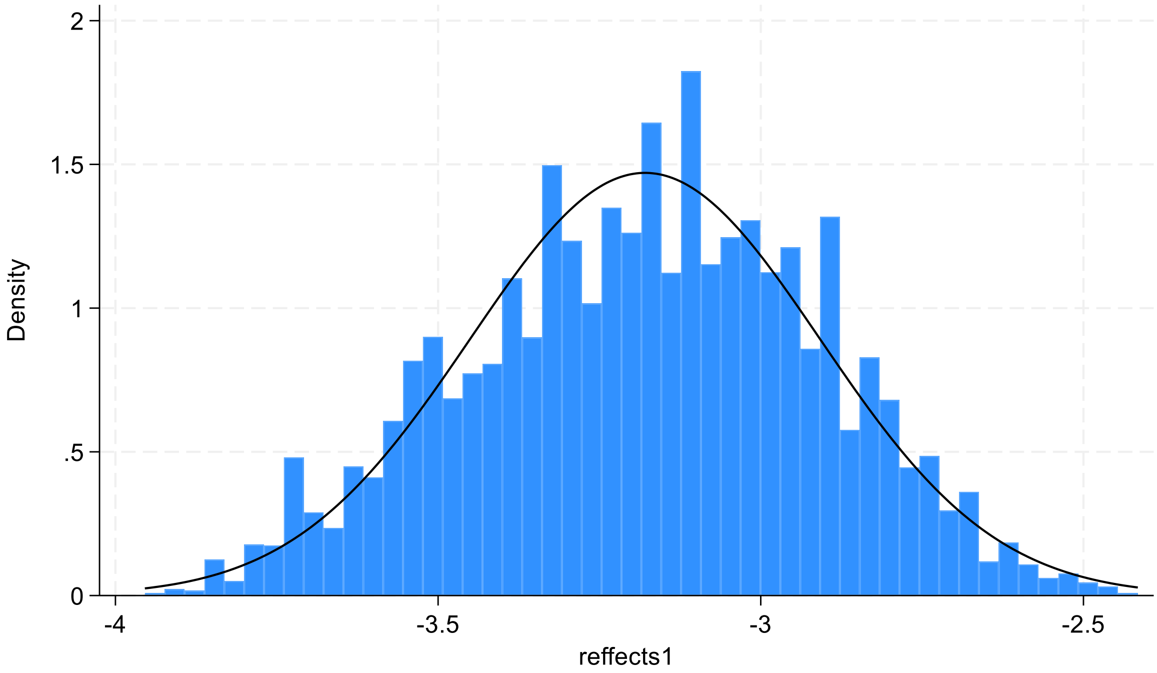

Figure 3: Histogram showing the normal distribution of random effects for ARI.

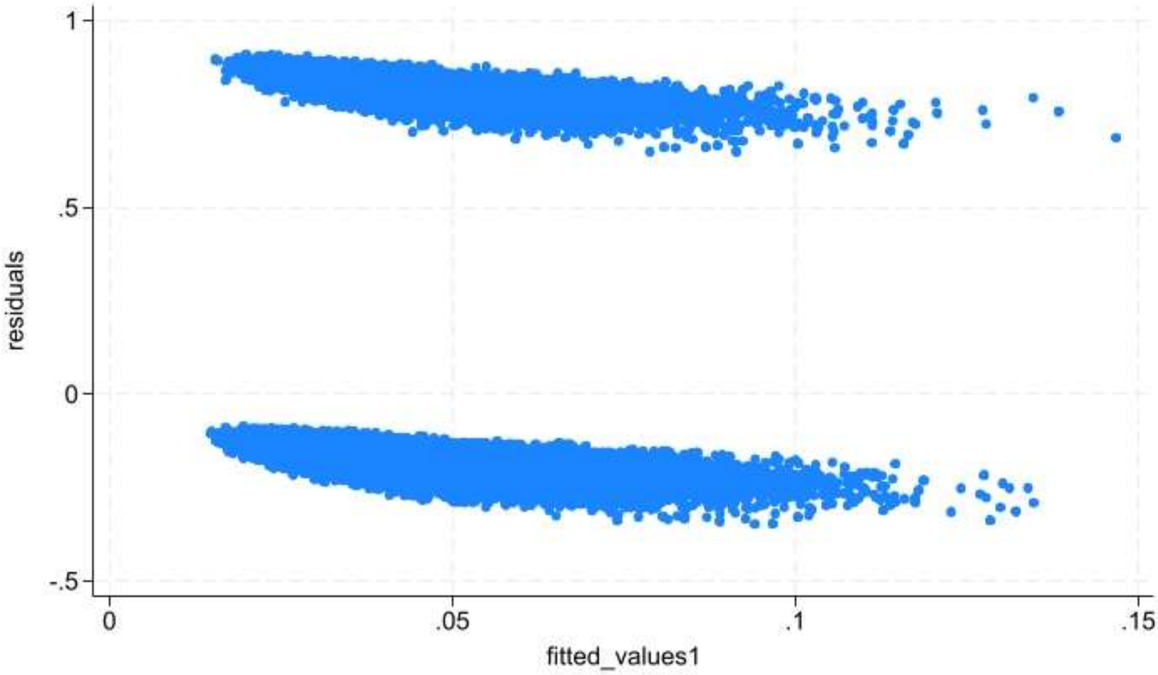

Figure 2: Scatter plot of residuals against fitted values for ARI.

Appendix 4: Prevalence of acute respiratory tract infections and diarrhoea among under five children in sub-Saharan Africa countries

Countries	ARI, % (95% CI)	Diarrhoea, % (95% CI)
Angola, DHS 2015-16	3.1 (2.7, 3.7)	15.8 (14.5, 17.2)
Benin, DHS 2017-18	2.9 (2.5, 3.3)	10.7 (9.9, 11.4)
Burundi, DHS 2016-17	7.2 (6.6, 7.9)	22.7 (21.6, 23.8)
Cameroon, DHS 2018	1.0 (0.8, 1.3)	12.4 (11.5, 13.4)
Chad, DHS 2014-15	7.7 (6.8, 8.8)	22.5 (21.2, 23.8)
Ethiopia, DHS 2015-16	6.7 (5.7, 7.9)	12.0 (10.8, 13.3)
Gambia, DHS 2019-20	4.8 (4.1, 5.6)	20.1 (18.8, 21.5)
Ghana, DHS 2014	3.7 (3.1, 4.4)	12.1 (10.8, 13.5)
Guinea, DHS 2018	2.1 (1.8, 2.6)	14.7 (13.5, 16.1)
Kenya, DHS 2014	8.7 (8.1, 9.2)	15.7 (14.8, 16.5)
Lesotho, DHS 2014	4.6 (3.7, 5.6)	12.4 (10.8, 14.3)
Liberia, DHS 2019-20	4.9 (3.8, 6.3)	16.9 (15.3, 18.5)
Madagascar, DHS 2021	2.5 (2.2, 2.9)	9.4 (8.7, 10.2)
Malawi, DHS 2015-16	5.5 (5.0, 6.0)	22.1 (21.1, 23.1)
Mali, DHS 2018	2.1 (1.6, 2.6)	17.4 (16.1, 18.8)
Mauritania, DHS 2019-21	4.2 (3.7, 4.9)	12.9 (11.9, 13.9)
Nigeria, DHS 2018	2.7 (2.4, 3.0)	13.0 (12.3, 13.7)
Rwanda, DHS 2019-20	1.8 (1.4, 2.1)	14.5 (13.6, 15.4)
Senegal, DHS 2019	5.2 (4.4, 6.0)	13.8 (12.4, 15.3)
Sierra Leone, DHS 2019	2.2 (1.7, 2.8)	7.4 (6.6, 8.3)
South Africa, DHS 2016	3.5 (2.6, 4.5)	11.6 (10.1, 13.3)
Tanzania, DHS 2015-16	3.9 (3.4, 4.5)	12.3 (11.4, 13.4)
Uganda, DHS 2016	9.8 (9.1, 10.5)	20.6 (19.6, 21.7)
Zambia, DHS 2018	1.8 (1.4, 2.2)	15.7 (14.6, 16.8)
Zimbabwe, DHS 2014-15	4.0 (3.4, 4.7)	17.6 (16.3, 19.1)
Pooled 25 SSA countries	4.6 (4.5, 5.8)	15.6 (15.3, 15.8)

Appendix 5: Prevalence of acute respiratory tract infections across study factors among children under five in sub-Saharan Africa countries, 2014-2021 (N = 253,166)

Variables	Male, n (%)	Female, n (%)	Total population, n (%)
**Child factors**			
Perceived baby birth size			
Below average	1035 (5.6)	1164 (5.1)	2199 (5.3)
Average or above average	4531 (4.3)	3956 (4.1)	8486 (4.2)
Early initiation of breastfeeding			
No	3591 (4.7)	3350 (4.5)	6941 (4.6)
Yes	2417 (4.7)	2146 (4.2)	4563 (4.5)
Duration of breastfeeding			
≤12 months	1594 (5.5)	1375 (4.9)	2968 (5.2)
>12 months	3958 (4.2)	3738 (4.1)	7696 (4.1)
**Maternal factors**			
Maternal age			
15-24 years	1621 (4.7)	1828 (5.1)	3450 (4.9)
25-34 years	2657 (4.4)	2878 (4.6)	5534 (4.5)
35+ years	1218 (4.1)	1302 (4.3)	2520 (4.2)
Birth order			
One	1233 (4,5)	1075 (4.0)	2308 (4.3)
2-4 Children	2862 (4.6)	2637 (4.4)	5500 (4.5)
5+ Children	1913 (5.0)	1784 (4.7)	3697 (4.9)
Maternal education			
No or low education	4593 (5.0)	4278 (4.7)	8870 (4.8)
Secondary or higher	1415 (4.0)	1218 (3.6)	2634 (3.8)
Maternal employment			
Not working	1437 (4.2)	1344 (4.0)	2782 (4.1)
Working	2029 (4.3)	1799 (3.9)	3829 (4.1)
Antenatal care			
Three or less visits	2149 (5.5)	1939 (5.1)	4088 (5.3)
4+ visits	2645 (5.1)	2305 (4.6)	4950 (4.9)
Place of birth			
Home	2260 (4.9)	2267 (4.9)	4527 (4.9)
Health facility	3746 (4.6)	3228 (4.1)	6974 (4.4)
**Household factors**			
Household wealth			
Poor	2916 (5.1)	2696 (4.8)	5612 (4.9)
Middle	1220 (4.8)	1114 (4.5)	2334 (4.6)
Rich	1872 (4.2)	1686 (3.8)	3558 (4.0)
Type of toilet system			
Not improved	3418 (5.2)	3131 (4.9)	6549 (5.0)
Improved	2481 (4.2)	2269 (3.9)	4750 (4.1)
Source of drinking water			
Not Protected	2642 (4.6)	2332 (4.1)	4975 (4.3)
Protected	3366 (4.8)	3163 (4.6)	6529 (4.7)
Type of cooking fuel			
Not cleaned	5395 (4.9)	4978 (4.6)	10373 (4.7)
Cleaned	605 (3.6)	511 (3.1)	1116 (3.3)

Appendix 6: Prevalence of diarrhoea across study factors among children under five in sub-Saharan Africa countries, 2014-2021 (N = 253,166)

Variables	Male, n (%)	Female, n (%)	Total population, n (%)
**Child factors**			
Perceived baby birth size			
Below average	3455 (18.8)	3969 (17.5)	7424 (18.1)
Average or above average	16348 (15.7)	13946 (14.3)	30294 (15.0)
Early initiation of breastfeeding			
No	11442 (15.0)	10213 (13.8)	21655 (14.4)
Yes	9175 (18.0)	8372 (16.6)	17548 (17.3)
Duration of breastfeeding			
≤12 months	5629 (19.4)	5081 (18.1)	10710 (18.8)
>12 months	14148 (15.2)	12809 (14.0)	26958 (14.6)
**Maternal factors**			
Maternal age			
15-24 years	6977 (19.6)	6369 (18.5)	13346 (19.1)
25-34 years	9539 (15.5)	8518 (14.1)	18057 (14.8)
35+ years	4101 (13.6)	3698 (12.4)	7799 (15.6)
Birth order			
One	4580 (16.8)	4146 (15.5)	8726 (16.2)
2-4 Children	9894 (15.9)	8859 (14.7)	18753 (15.3)
5+ Children	6143 (16.2)	5581 (14.9)	11724 (15.5)
Maternal education			
No or low education	15412 (16.8)	14159 (15.6)	29572 (16.2)
Secondary or higher	5205 (14.8)	4426 (13.1)	9631 (13.9)
Maternal employment			
Not working	5316 (15.7)	4790 (14.5)	10107 (15.1)
Working	7499 (15.9)	6673 (14.6)	14172 (15.3)
Antenatal care visits			
Three or less visits	7587 (19.5)	6944 (18.3)	14531 (18.9)
4+ visits	9598 (18.7)	8374 (16.8)	17972 (17.8)
Place of birth			
Home	14233 (15.5)	6895 (15.0)	14233 (15.5)
Health facility	24963 (15.6)	11687 (14.9)	24963 (15.6)
**Household factors**			
Household wealth			
Poor	9823 (17.3)	8951 (16.0)	18773 (16.7)
Middle	4178 (16.4)	3652 (14.7)	7829 (15.5)
Rich	6616 (14.8)	5984 (13.6)	12600 (14.2)
Type of toilet system			
Not improved	10913 (16.7)	9963 (15.6)	20876 (16.2)
Improved	9227 (15.7)	8230 (14.3)	17457 (15.0)
Source of drinking water			
Not Protected	9146 (16.0)	8347 (14.8)	17493 (15.4)
Protected	11471 (16.4)	10239 (15.0)	21710 (15.7)
Type of cooking fuel			
Not cleaned	18374 (16.7)	2048 (12.5)	34886 (16.0)
Cleaned	2218 (13.2)	16512 (15.3)	4266 (12.9)
**Community level factors**			
Place of residence			
Urban	5866 (14.8)	5332 (13.8)	11198 (14.3)
Rural	14751 (16.8)	13254 (15.40)	28003 (16.1)

Appendix 7: DHS data access grant letter

Oct 24, 2022

Kedir Yimam Ahmed
Samara University
Ethiopia
Request Date: 10/24/2022

Dear Kedir Yimam Ahmed:

This is to confirm that you are approved to use the following Survey Datasets for your registered research paper titled: "Determinants of childhood stunting in Sub-Saharan Africa ":

Angola, Benin, Botswana, Burkina Faso, Burundi, Cameroon, Central African Republic, Chad, Comoros, Congo, Congo Democratic Republic, Cote d'Ivoire, Eritrea, Eswatini, Ethiopia, Gabon, Gambia, Ghana, Guinea, Kenya, Lesotho, Liberia, Madagascar, Malawi, Mali, Mauritania, Mozambique, Namibia, Niger, Nigeria, Nigeria (Ondo State), Rwanda, Sao Tome and Principe, Senegal, Sierra Leone, South Africa, Sudan, Tanzania, Togo, Uganda, Zambia, Zimbabwe

For restricted surveys, you must also request special permission from the Implementing Agencies. If approved, the restricted datasets will be provided to you by FTP.

To access the datasets, please login at: https://www.dhsprogram.com/data/dataset_admin/login_main.cfm. The user name is the registered email address, and the password is the one selected during registration.

The IRB-approved procedures for DHS public-use datasets do not in any way allow respondents, households, or sample communities to be identified. There are no names of individuals or household addresses in the data files. The geographic identifiers only go down to the regional level (where regions are typically very large geographical areas encompassing several states/provinces). Each enumeration area (Primary Sampling Unit) has a PSU number in the data file, but the PSU numbers do not have any labels to indicate their names or locations. In surveys that collect GIS coordinates in the field, the coordinates are only for the enumeration area (EA) as a whole, and not for individual households, and the measured coordinates are randomly displaced within a large geographic area so that specific enumeration areas cannot be identified.

The DHS Data may be used only for the purpose of statistical reporting and analysis, and only for your registered research. To use the data for another purpose, a new research project must be registered. All DHS data should be treated as confidential, and no effort should be made to identify any household or individual respondent interviewed in the survey. Also, be aware that re-distribution of any DHS micro-level data, either directly or within any tool/dashboard, is not permitted. Please reference the complete terms of use at: https://dhsprogram.com/Data/terms-of-use.cfm.

The data must not be passed on to other researchers without the written consent of DHS. However, if you have coresearchers registered in your account for this research paper, you are authorized to share the data with them. All data users are required to submit an electronic copy (pdf) of any reports/publications resulting from using the DHS data files to: references@dhsprogram.com.

Sincerely,

Bridgette Wellington

Bridgette Wellington
Data Archivist
The Demographic and Health Surveys (DHS) Program

530 Gaither Road, Suite 500, Rockville, MD 20850 USA +1.301.407.6500 +1.301.407.6501 fax icf.com

References

1. Ahmed KY, Agho KE, Page A, Arora A, Ogbo FA. Mapping Geographical Differences and Examining the Determinants of Childhood Stunting in Ethiopia: A Bayesian Geostatistical Analysis. *Nutrients* 2021; **13**(6).
2. Ahmed KY, Page A, Arora A, Ogbo FA. Associations between infant and young child feeding practices and acute respiratory infection and diarrhoea in Ethiopia: A propensity score matching approach. *PloS one* 2020; **15**(4): e0230978.
3. Ogbo FA, Page A, Idoko J, Agho KE. Population attributable risk of key modifiable risk factors associated with non-exclusive breastfeeding in Nigeria. *BMC public health* 2018; **18**(1): 247.
4. Ahmed KY, Dadi AF, Ogbo FA, et al. Population-Modifiable Risk Factors Associated With Childhood Stunting in Sub-Saharan Africa. *JAMA Network Open* 2023; **6**(10): e2338321-e.
5. Ahmed KY, Agho KE, Page A, et al. Mapping Geographical Differences and Examining the Determinants of Childhood Stunting in Ethiopia: A Bayesian Geostatistical Analysis. *Nutrients* 2021; **13**(6).
6. Ahmed KY, Page A, Arora A, Ogbo FA, Global M, Child Health Research c. Associations between infant and young child feeding practices and acute respiratory infection and diarrhoea in Ethiopia: A propensity score matching approach. *PloS one* 2020; **15**(4): e0230978.
7. Ahmed KY, Ross AG, Hussien SM, Agho KE, Olusanya BO, Ogbo FA. Mapping Local Variations and the Determinants of Childhood Stunting in Nigeria. *International journal of environmental research and public health*, 2023. (accessed.
8. Croft, Trevor N, Aileen MJM, Courtney KA, et al. Guide to DHS Statistics: DHS-7. Rockville, Maryland, USA; 2018.
9. Ahmed KY, Abrha S, Page A, et al. Trends and determinants of underweight and overweight/obesity among urban Ethiopian women from 2000 to 2016. *BMC public health* 2020; **20**(1): 1276.
10. Ahmed KY, Rwabilimbo AG, Abrha S, et al. Factors associated with underweight, overweight, and obesity in reproductive age Tanzanian women. *PloS one* 2020; **15**(8): e0237720.
11. The DHS Program. Wealth Index Construction. 2016. https://www.dhsprogram.com/topics/wealth-index/Wealth-Index-Construction.cfm.
12. Naz S, Page A, Agho KE. Household Air Pollution and Under-Five Mortality in Bangladesh (2004-2011). *International journal of environmental research and public health* 2015; **12**(10): 12847-62.
13. Naz S, Page A, Agho KE. Household air pollution from use of cooking fuel and under-five mortality: The role of breastfeeding status and kitchen location in Pakistan. *PloS one* 2017; **12**(3): e0173256-e.
14. Girma M, Hussein A, Norris T, et al. Progress in Water, Sanitation and Hygiene (WASH) coverage and potential contribution to the decline in diarrhea and stunting in Ethiopia. *Maternal & child nutrition* 2021: 1.
15. Miettinen OS. Proportion of disease caused or prevented by a given exposure, trait or intervention. *American journal of epidemiology* 1974; **99**(5): 325-32.
16. Ahmad K, Rasmus Oestergaard N, Mohammad Ali M. Methods matter: population attributable fraction (PAF) in sport and exercise medicine. *British Journal of Sports Medicine* 2020; **54**(17): 1049.
17. Khosravi A, Nazemipour M, Shinozaki T, Mansournia MA. Population attributable fraction in textbooks: Time to revise. *Global Epidemiology* 2021; **3**: 100062.
18. Wilson LF, Page AN, Dunn NAM, Pandeya N, Protani MM, Taylor RJ. Population attributable risk of modifiable risk factors associated with invasive breast cancer in women aged 45–69 years in Queensland, Australia. *Maturitas* 2013; **76**(4): 370-6.
